# Angiotensin type-1 receptor and ACE2 autoantibodies in Parkinson´s disease

**DOI:** 10.1038/s41531-022-00340-9

**Published:** 2022-06-14

**Authors:** Carmen M. Labandeira, Maria A. Pedrosa, Aloia Quijano, Rita Valenzuela, Pablo Garrido-Gil, Mariña Sanchez-Andrade, Juan A. Suarez-Quintanilla, Ana I. Rodriguez-Perez, Jose L. Labandeira-Garcia

**Affiliations:** 1 Research Center for Molecular Medicine and Chronic diseases (CIMUS), IDIS, University of Santiago de Compostela, Santiago de Compostela, Spain; 2 Neurology Service, Hospital Alvaro Cunqueiro, University Hospital Complex, Vigo, Spain. Neurology Service. University Hospital of Ourense, Ourense, Spain; 3 Networking Research Center on Neurodegenerative Diseases (CIBERNED), Madrid, Spain; 4 Obstetric Service, University Clinical Hospital of Santiago de Compostela, Santiago de Compostela, Spain; 5 Primary Health-Care Unit Fontiñas, IDIS, Santiago de Compostela, Spain

**Keywords:** Parkinson's disease, Translational research

## Abstract

The role of autoimmunity in neurodegeneration has been increasingly suggested. The renin-angiotensin system (RAS) autoantibodies play a major role in several peripheral inflammatory processes. Dysregulation of brain RAS has been involved in neuroinflammation and neurodegeneration. We aimed to know whether angiotensin type-1 receptor (AT1) autoantibodies (AT1 agonists) and angiotensin-converting enzyme 2 (ACE2) autoantibodies (ACE2 antagonists) may be involved in Parkinson’s disease (PD) progression and constitute a new therapeutical target. Both AT1 and ACE2 serum autoantibodies were higher in a group of 117 PD patients than in a group of 106 controls. Serum AT1 autoantibodies correlated with several cytokines, particularly Tumor Necrosis Factor Ligand Superfamily Member 14 (TNFSF14, LIGHT), and 27-hydroxycholesterol levels. Serum ACE2 autoantibodies correlated with AT1 autoantibodies. Both autoantibodies were found in cerebrospinal fluid (CSF) of four PD patients with CSF samples. Consistent with the observations in patients, experimental dopaminergic degeneration, induced by 6-hydroxydopamine, increased levels of autoantibodies in serum and CSF in rats, as well as LIGHT levels and transglutaminase activity in rat substantia nigra. In cultures, administration of AT1 autoantibodies enhanced dopaminergic neuron degeneration and increased levels of neuroinflammation markers, which was inhibited by the AT1 antagonist candesartan. The results suggest dysregulation of RAS autoantibodies as a new mechanism that can contribute to PD progression. Therapeutical strategies blocking the production, or the effects of these autoantibodies may be useful for PD treatment, and the results further support repurposing AT1 blockers (ARBs) as treatment against PD progression.

## Introduction

The possible role of autoimmune processes in initiation and/or progression of neurodegeneration, and particularly Parkinson´s disease (PD), has been increasingly suggested^1,2^. Different brain disorders, including neurodegenerative diseases, have been associated with autoantibodies that target the extracellular domains of neuronal or glial proteins^3,4^. These autoantibodies may enter cerebrospinal fluid (CSF) and brain after crossing the blood-brain barrier (BBB), particularly a permeable BBB, although an increasing number of studies suggest their intrathecal synthesis^3,5^. Antibodies against G Protein-Coupled Receptors (GPCRs) are particularly interesting, as they can bind the corresponding receptors and play agonist-stimulatory or antagonist-inhibitory effects on GPCRs, affecting neuronal and glial homeostatic regulation of GPCRs^6,7^, but dysregulations may lead to functional alterations or disease^8^.

The renin-angiotensin system (RAS) has been involved in the progression of PD and other neurodegenerative diseases by promotion of oxidative stress and neuroinflammation^9,10^. Interestingly, a recent study shows that the population of dopaminergic neurons most vulnerable to degeneration in PD can be identified by their high expression of angiotensin type-1 receptor (AT1) gen^11^. The tissue RAS, including brain RAS, consists of two arms that counteract each other: a proinflammatory and pro-oxidative axis mainly formed by angiotensin II (AngII) and AT1 receptors, and an anti-inflammatory and anti-oxidative axis composed by AngII/AT2 receptors together with Ang1-7 /Mas receptors (MasR)^12,13^. Angiotensin-converting enzyme 2 (ACE2) converts peptides of the proinflammatory axis into peptides of the anti-inflammatory axis. The major role of the tissue RAS in inflammatory responses has been recently shown in COVID-19 pandemic, being ACE2 the entry receptor for SARS-CoV-2 and a key component for COVID-19 progression^14^. The use of AT1 antagonists such as candesartan for neuroprotection has been proposed from results in experimental models and clinical trials^15,16^. Interestingly, activation of endothelial AT1 receptors plays a key role in BBB disruption^17,18^. However, the possible involvement of autoimmune responses targeting RAS components in the progression of PD has not been investigated.

Agonistic autoantibodies for AT1 receptors (AT1-AA) play a major role in peripheral processes such as preeclampsia^19^, malign hypertension^20^, and other inflammation-related diseases^21,22^. In peripheral tissues, AT1-AA activate AT1 receptors, as agonist autoantibodies. Furthermore, they stabilize AT1 in a permanent activated state and upregulate AT1 receptor expression by inhibiting their internalization thus amplifying the RAS proinflammatory axis activity^23–25^. ACE2 autoantibodies (ACE2-AA) have antagonist effects on ACE2 function, therefore inhibiting the anti-inflammatory axis, and promoting, as in the case of AT1-AA, the proinflammatory response^26,27^. Recently, increased serum levels of AT1-AA and ACE2-AA were associated to severity of COVID-19 outcome, possibly by enhancing the tissue inflammatory response^28–30^.

Although different studies have involved dysregulation of brain RAS in the progression of PD, the mechanisms involved in brain RAS dysregulation have not been clarified, and the possible role of AT1-AA and ACE2-AA has not been investigated. In the present study we used a cohort of PD patients and non-PD controls as well as PD animal and in vitro models to study the possible role of autoantibodies targeting AT1 and ACE2 in the progression of PD.

## Results

### Patient characteristics

A total of 106 controls (mean age: 64.9 years old ± 9.25 Standard Deviation, SD, being 51 men and 55 women) and 117 PD patients (mean age: 69.43 years old ± 10.09 SD; being 57 men and 60 women) were enrolled in the study (Supplementary Table 1). Baseline characteristics from the PD cohort are shown in Supplementary Table 2. Inclusion criteria and sample collection are described in the Methods section.

### AT1-AA and ACE2-AA in Parkinson´s disease patients

**Fig. 1:**
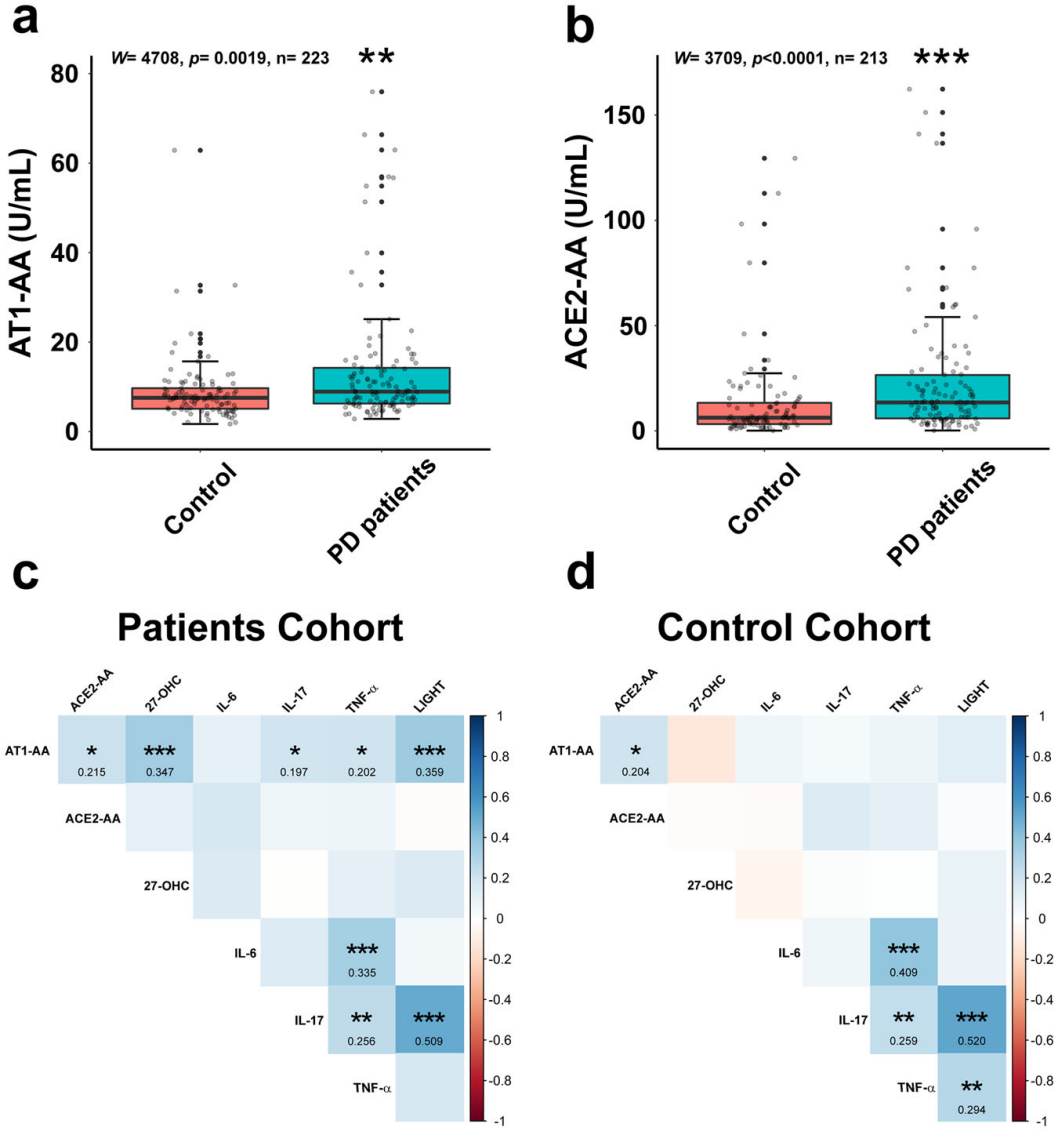
Autoantibody serum levels and their correlations with inflammatory cytokines and 27-hydroxycholesterol in controls and PD patients. Parkinson´s disease (PD) patients had significantly higher serum levels of AT1-AA (**a**; Wilcoxon test, *W* = 4708, *P* = 0.0019) and ACE2-AA (**b**; Wilcoxon test, W = 3709; *P* < 0.0001) than controls. Results of Spearman’s correlation matrix show variables (AT1-AA, ACE2-AA, 27-OHC, cytokines) correlating at varying levels of significance (*P < 0.05; **P < 0.01; ***P < 0.001) in PD patients (**c**) and controls (**d**). In **a** and **b**, data distribution is shown using a box plot with boxes representing the IQR and the median (black line) and whiskers representing ± 1.5 IQR. IQR Interquartile range. **P < 0.01; ***P < 0.001. 27-OHC, 27-Hydroxycholesterol; AT1-AA: Autoantibodies for AT1 receptors; ACE2-AA: ACE2 Autoantibodies.

Median of AT1-AA serum concentrations were 7.556 [Interquartile range (IQR) 5.109–9.699] U/mL for the control group and 8.923 [IQR 6.271–14.240] U/mL for PD patients. Median of ACE2-AA serum concentrations were 6.305 [IQR 3.265–13.349] U/mL in the control group and 13.519 [IQR 5.974–26.576] U/mL for PD patients. Wilcoxon-Mann-Whitney test analysis revealed significantly higher serum levels for AT1-AA (*P* = 0.0019; *W* = 4708) and for ACE2-AA (*P* < 0.0001; *W* = 3709) in the PD group than in the control group (Fig. 1a, b). Complementary analysis excluding controls and patients under immunotherapy (ATI-AA: *P* = 0.0008, *W* = 3695; ACE2-AA: *P* = 0.0001, *W* = 3023) or ACEIs/ARAII (ATI-AA: *P* = 0.0002, *W* = 2200; ACE2-AA: P = 0.0004, *W* = 2016) also resulted significant differences (Supplementary Fig. 1).

A logistic regression was performed to estimate the association between PD and autoantibodies. Furthermore, variables such as sex, age, immunosuppressive treatment, ACEI, ARAII, smoking habits and heart disease were also included as independent variables in order to account for possible confounding associations. The inclusion of the different predictors was assessed via stepwise model selection using the minimum Akaike information criterion. This resulted in a final logistic model that selected age, angiotensin-converting enzyme inhibitors (ACEIs) and immunosuppressive treatment. The model’s explanatory power is moderate (Precision = 0.64). Interestingly, both AT1-AA and age indicate positive association with PD. The odds of having a patient diagnosed with PD is increased 7.4% (Odds Ratio [OD] = 1.07, 95% Confidence Interval [CI] = 1.03–1.13) and 6.4% (OR = 1.06, 95%CI = 1.03–1.10) per unit of concentration and year, respectively.

Analogous logistic model was performed for ACE2-AA, age, ACEIs and immunosuppressive treatment. The model’s explanatory power is moderate (Precision = 0.65). The odds of having a patient diagnosed with PD is increased 1.9% (Odds Ratio [OD] = 1.02, 95% Confidence Interval [CI] = 1.01–1.04) and 4.7% (OR = 1.05, 95%CI = 1.02–1.08) per unit of concentration and year, respectively. Characteristics of models are shown in Tables 1 and 2. Moreover, AT1-AA and ACE2-AA are also statistically significant after excluding controls and patients with immunosuppressive treatment (OR AT1-AA(Adjusted) = 1.12, OR ACE2-AA(Adjusted) = 1.02). When excluding patients treated with ACEIs, the odds of AT1-AA and ACE2-AA are also higher than 1.

**Table 1:** Results for the AT1-AA Multivariate Logistic Regression Model.

	Estimate	SE	Z VALUE	PR(>| z | )	OR	2.5%	97.5%
(INTERCEPT)	−4.4866976	1.1829256	−3.792882	0.0001489	0.0112578	0.0010071	0.1060520
AT1-AA	0.0716248	0.0229940	3.114934	0.0018399	1.0742522	1.0334422	1.1328496
AGE	0.0625031	0.0166368	3.756917	0.0001720	1.0644977	1.0314015	1.1012257
ACEi	−1.0825012	0.4732857	−2.287205	0.0221839	0.3387472	0.1300229	0.8448410
Immunosuppressive treatment	−1.5811719	0.6432487	−2.458104	0.0139673	0.2057339	0.0502494	0.6644599

*AT1-AA* Autoantibodies for AT1 receptors, *ACEi* Angiotensin-converting enzyme inhibitors.

**Table 2:** Results for the ACE2-AA Multivariate Logistic Regression Model.

	Estimate	SE	Z VALUE	PR( > | z | )	OR	2.5%	97.5%
(INTERCEPT)	−2.9376328	1.0681633	−2.750172	0.0059564	0.0529910	0.0060951	0.4097122
ACE2-AA	0.0187801	0.0082492	2.276580	0.0228113	1.0189575	1.0051301	1.0380461
AGE	0.0460548	0.0160649	2.866799	0.0041465	1.0471318	1.0154659	1.0817985
ACEi	−0.9748823	0.4681841	−2.082263	0.0373185	0.3772367	0.1465484	0.9334512
Immunosuppressive treatment	−1.5960436	0.6194379	−2.576600	0.0099777	0.2026969	0.0522467	0.6270842

*ACE2-AA* ACE2 Autoantibodies, *ACEi* Angiotensin-converting enzyme inhibitors.

### Relationships between serum AT1-AA and ACE2-AA, cytokines, and 27-Hydroxycholesterol

Spearman correlations were used to study possible relationship between AT1-AA and ACE2-AA. In addition, Spearman correlations were also used to observe monotonous increasing or decreasing relationships of serum levels of autoantibodies with interleukins and 27-hydroxycholesterol serum levels. We analysed those compounds (TNFSF14, TNF-α, IL-6, IL-17) that have been associated to angiotensin autoantibodies in previous studies on other diseases in peripheral tissues, or that have been correlated with brain RAS neurodegenerative effects (27-hydroxycholesterol) (see Discussion). In PD patients, the results showed a positive and significant correlation between serum levels of AT1-AA and ACE2-AA (*ρ* = 0.215, *P* < 0.05). There were also significant positive correlations between serum levels of AT1-AA and LIGHT (TNFSF14; *ρ* = 0.359, *P* < 0.001), 27-Hydroxycholesterol (*ρ* = 0.347, *P* < 0.001), IL-17 (*ρ* = 0.197, *P* < 0.05) and TNF-α (*ρ* = 0.202, *P* < 0.05). Interestingly, we found a strong and significant correlation between IL-17 and LIGHT serum levels (*ρ* = 0.509, *P* < 0.001). Significant correlations were also observed between TNF-α and IL-6 (*ρ* = 0.335, *P* < 0.001) and IL-17 (*ρ* = 0.256, *P* < 0.001) serum levels. However, no correlation was found between serum levels of ACE2-AA and the studied set of interleukins or 27-Hydroxycholesterol (Fig. 1c).

In the control cohort, we also observed significant correlation between serum levels of AT1-AA and ACE2-AA (*ρ* = 0.204, *P* < 0.05), as well as between TNF-α and IL-6 (*ρ* = 0.409, *P* < 0.001) and IL-17 (*ρ* = 0.259, *P* < 0.01), and between LIGHT and IL-17 (*ρ* = 0.520, *P* < 0.001) or TNF-α serum levels (*ρ* = 0.294, *P* < 0.01) (Fig. 1d).

### Presence of AT1-AA and ACE2-AA in CSF in four PD patients of our cohort with CSF samples

Among PD patients recruited in this study, only 4 required lumbar punctures for other diagnostic purposes, showing no pathological changes as detected by routine parameters. Their CSF was used to determine the presence of AT1-AA and ACE2-AA. Both autoantibodies were found in the CSF of these 4 patients and the concentration, estimated as mean, was 4.15 × 10^−2^ U/mL ± 0.3 × 10^−2^ (SD) for AT1-AA and 7.82 × 10^−2^U/mL ± 4.92 × 10^−2^ (SD) for ACE2-AA. Although the n is too small for comparative quantitative studies, the levels of autoantibodies in CSF in 5 healthy non-PD patients of our cohort were 2.32 × 10^−2^ U/mL ± 0.538 × 10^−2^ (SD) for AT1-AA, and 4.43 × 10^−2^ U/ mL ± 3.37 × 10^−2^ (SD) for ACE2-AA. Data from these 4 patients, cannot be representative of the overall PD population. However, CSF samples from PD patients without any additional pathology that may affect CSF are extremely difficult to obtain, and we think that these data, although insufficient to be generalized to the PD population, should be mentioned with all cautions, because provide information that agree with the observations in the animal models (see below).

To determine possible intrathecal synthesis of autoantibodies in the analyzed PD patients, we calculated the corrected antibody index (AI) as AI = Q_AT1-AA_/Q_IgG-Total_ and AI = Q_ACE2-AA_/Q_IgG-Total_^31,32^. The results for AI were 10.958 [IQR 7.296– 15.454] delimited by a minimum and a maximum value of 4.687 and 18.899 for AT1-AA and 6.110 [IQR 3.803– 7.809] with a minimum of 2.355 and a maximum of 8.649 for ACE2-AA. Therefore, the four PD patients with CSF samples showed AI higher than 1.5, which is indicative of intrathecal synthesis. Again, the data of these 4 patients cannot be generalized to all PD patients. However, the observation reveals that intrathecal synthesis can take place at least in some PD patients.

### 6-OHDA-induced dopaminergic degeneration induces serum and CSF AT1-AA and ACE2-AA

**Fig. 2:**
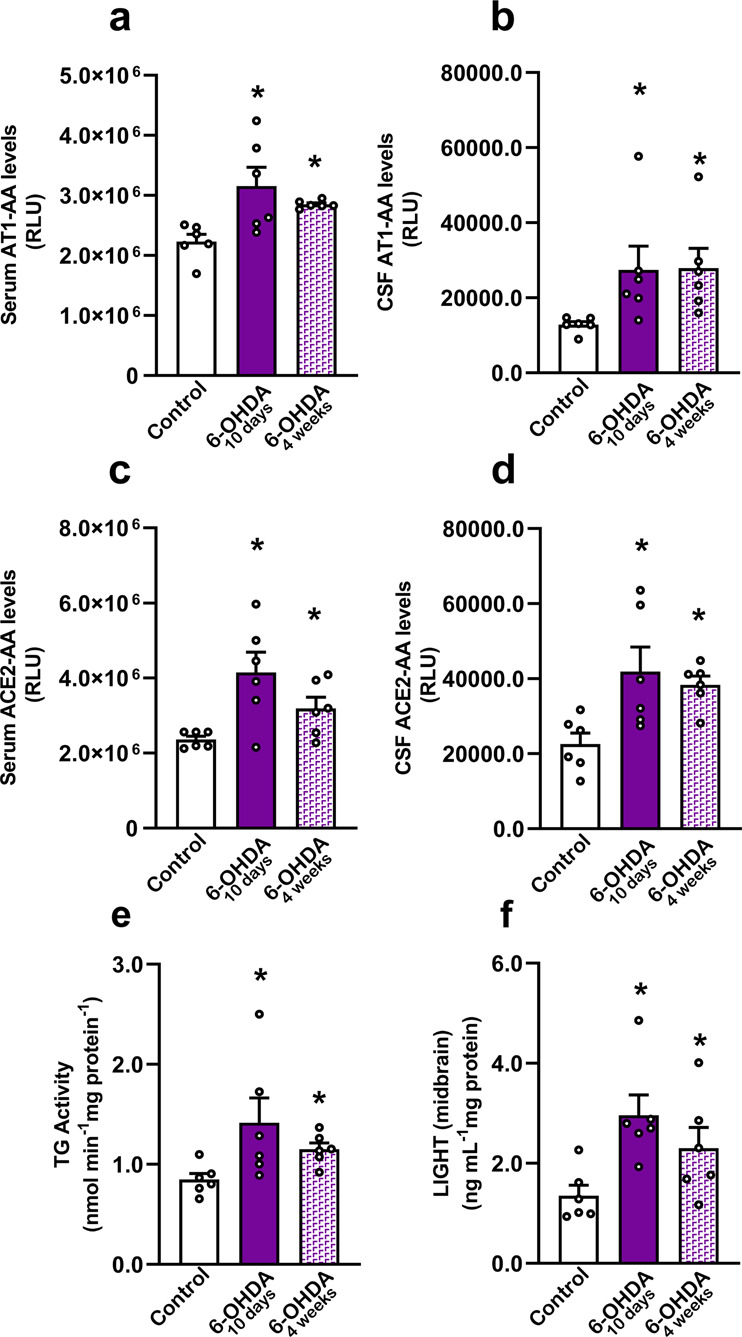
Increase in serum and CSF AT1-AA and ACE2-AA levels induced by dopaminergic degeneration in rats. Serum levels of AT1-AA (**a**) and ACE2-AA (**c**) 10 days and 4 weeks after 6-OHDA injection (i.e. in the acute period of degeneration and neuroinflammation and when the lesion is stabilized and neuroinflammation has decreased, respectively). A significant increase in AT1-AA (**b**) and ACE2-AA (**d**) is observed in CSF 10 days and 4 weeks after 6-OHDA injection. A significant increase in levels of LIGHT cytokine (**f**) and transglutaminase activity (**e**) is observed in the nigral region 10 days and 4 weeks after 6-OHDA lesion, which may contribute to the increase in autoantibody production. Data are given as means ± SEM. *P < 0 .05 compared to the control group. Kruskal–Wallis One Way Analysis of Variance on Ranks with Student-Newman-Keuls Method post hoc test (**a**–**c**, **e**, **f**) and one-way ANOVA followed by the Student–Newman–Keuls Method for multiple comparisons (**d**). AT1-AA: Autoantibodies for AT1 receptors; ACE2-AA: ACE2 Autoantibodies.

The results observed in PD patients suggest that both AT1-AA and ACE2-AA may be induced by dopaminergic degeneration. To confirm this issue in PD models, we injected rats with 6-OHDA to know if the experimentally induced dopaminergic cell death and/or the accompanying neuroinflammatory process may induce AT1-AA and ACE2-AA. We analyzed serum and CSF from short term (10 days, i.e. within the acute period of neurodegeneration and neuroinflammation) and long-term (4 weeks; i.e. when the neurodegeneration is stabilized and neuroinflammation has decreased) 6-OHDA lesioned rats, and we found a significant increase in serum levels of AT1-AA and ACE2-AA both at 10 days and 4-weeks post-lesion (Fig. 2a, c). In the CSF, we also found an increase in levels of AT1-AA and ACE2-AA at 10 days and 4 weeks after 6-OHDA administration (Fig. 2b, d). This suggests that the autoantibody production is related to neuron death and neuroinflammation. In PD, an initial death of dopaminergic neurons and/or the corresponding neuroinflammatory process may induce AT1-AA and ACE2-AA, which may contribute to progression of the disease during the long period of active cell death and neuroinflammation. Finally, we studied possible mechanisms involved in neoantigen production by analyzing levels of LIGHT cytokine (TNFSF14) and transglutaminases in the nigral region 10 days and 4 weeks after 6-OHDA lesion. A significant increase in LIGHT levels and transglutaminase activity was observed 10 days and 4 weeks after the dopaminergic lesion (Fig. 2e, f).

To further support the observations of the present study in the 6-OHDA model of dopaminergic degeneration, we analyzed stored serum samples from a previous study using a different and more chronic model of dopaminergic degeneration induced by administration of neurospecific adeno-associated viral vectors serotype 9 coding for a mutated form of human alpha-synuclein (SynA53T). All details on methods and characteristics of the used animals can be found in our previous study^16^. As in the case of the 6-OHDA model used in the present study, we observed a significant increase in levels of autoantibodies (Supplementary Fig. 2).

### AT1-AA enhance dopaminergic neuron death in cell cultures

**Fig. 3:**
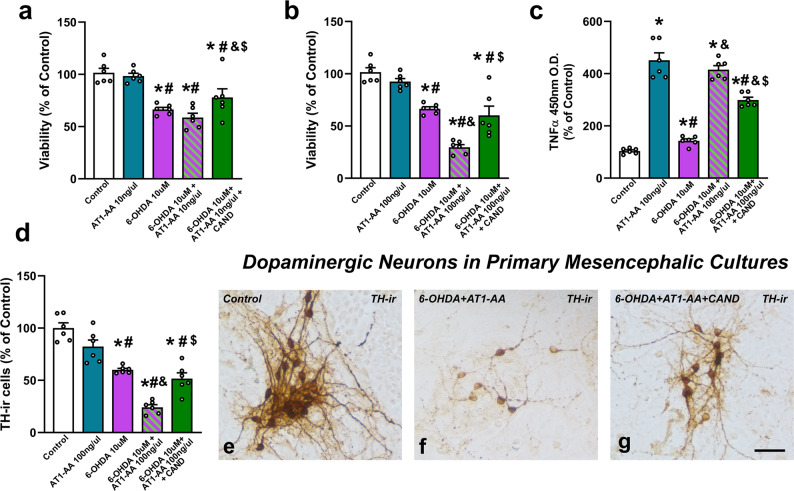
Primary mesencephalic cultures showing that AT1-AA enhance the deleterious effects of 6-OHDA (10 µM). In 6-OHDA-treated cultures, deleterious effects on cell viability are not significant with a dose of 10 ng/µl AT1-AA (i.e. 10 µM 6-OHDA + 10 ng/µl AT1-AA) (**a**) but are significant at 100 ng/µl (i.e. 10 µM 6-OHDA + 100 ng/µl AT1-AA), as revealed by the MTT assay (**b)** and TH-positive neuron counting **(d**), which was inhibited by the AT1 receptor blocker candesartan. AT1-AA alone did not induce significant decrease in cell viability (**a, b, d**). Interestingly, AT1-AA administration (100 ng/µl) also induced a marked increase in levels of TNF-α in cultures, which is significantly reduced by treatment with candesartan (**c**). Photomicrographs of dopaminergic (TH-ir) neurons in control cultures (**e**), cultures treated with AT1-AA plus 6-OHDA (**f**) and cultures treated with AT1-AA plus 6-OHDA plus candesartan (**g**). Scale bar 25 µm. Data are given as means ± SEM. *P < 0 .05 compared to the corresponding control group, ^#^*P* < 0.05 compared to AT1-AA group; ^&^*P* < 0.05 compared to 6-OHDA group; ^$^*P* < 0.05 compared to 6-OHDA + AT1-AA group. Kruskal–Wallis One Way Analysis of Variance on Ranks with Student-Newman-Keuls Method post hoc test. AT1-AA: Autoantibodies for AT1 receptors; CAND: Candesartan; 6-OHDA: 6-Hydroxydopamine; TH-ir: TH-immunoreactive.

As autoantibodies were detected in the CSF, we treated rat primary neuron-glia mesencephalic cultures with AT1-AA to know if they may enhance dopaminergic neuron death triggered by a low dose (10 µM) of the dopaminergic neurotoxin 6-OHDA. AT1-AA (10 and 100 ng/µl) were simultaneously administered to cultures, and we observed a significant decrease in dopaminergic neuron viability with doses of 100 ng/µl, which was inhibited by treatment with the AT1 antagonist candesartan, confirming that AT1-AA can enhance dopaminergic degeneration *via* AT1 receptor overactivation (Fig. 3). As previously observed in primary cultures treated with Ang II alone^33^, the neuron death induced by AT1-AA alone was not significantly different with the present AT1-AA doses; however, AT1-AA (100 ng/µl) induced a significant increase in cell death induced by a low dose of the dopaminergic neurotoxin as revealed by the MTT assay (Fig. 3b). The deleterious effect on dopaminergic neuron death was confirmed by counting the number of TH-immunoreactive neurons in cultures that received different treatments. Again, we observed that treatment with AT1-AA (100 ng/µl) significantly increased the dopaminergic neuron death triggered by 10 µM 6-OHDA (Fig. 3d–g). Consistent with the enhancing effect of AT1-AA on cell death, AT1-AA administration induced a marked increase in culture levels of TNF-α, a key mediator of dopaminergic neurodegeneration^1^ (Fig. 3c), probably related to microglial activation, which plays a major role in Ang II-induced dopaminergic neuron death, as we previously shown after AT1 stimulation with Ang II^33,34^.

**Fig. 4:**
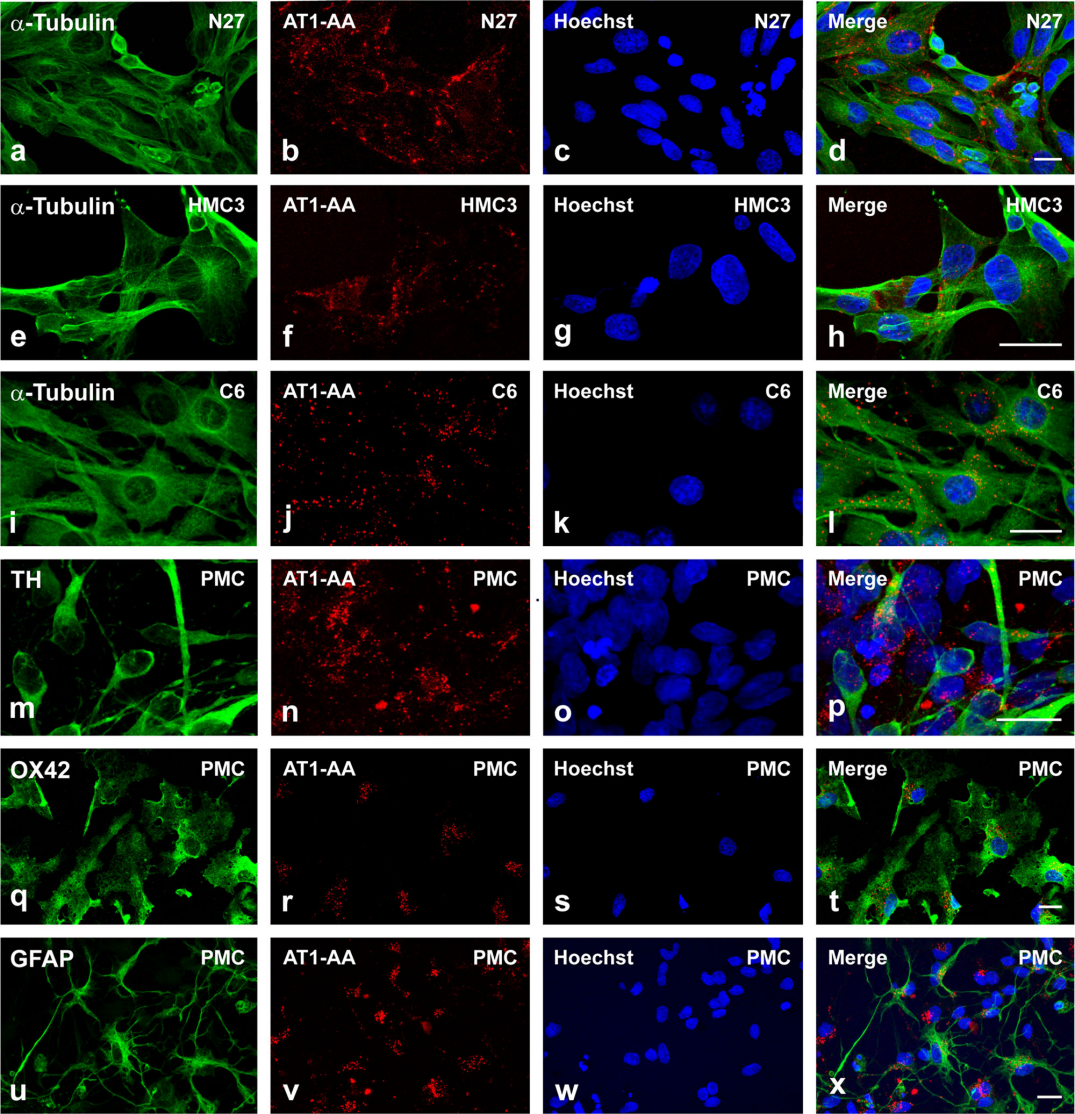
Dopaminergic neurons, astrocytes and microglia bearing fluorescent AT1-AA. Triple fluorescent labelling for (green) the cell marker anti-α-tubulin (**a, e, i**), the dopaminergic neuron marker Tyrosine hydroxylase (TH; **m**), the microglial marker CD11b (OX42; **q**), the astroglial marker GFAP(**u**), and the nuclear marker Hoechst 33342 (blue), and AT1-AA (red dots) in cell lines of dopaminergic neurons (N27; **a**–**d**), microglia (HMC3; **e**–**h**), astroglia (C6; **i**–**l**), and primary mesencephalic cultures (PMC; **m**–**x**) containing dopaminergic neurons (**m**–**p**), microglia (**q**–**t**) and astrocytes (**u**–**x**). Labelling for AT1-AA (red dots) is observed at the cell surface, with practically no colocalization (yellow) with the cytoplasmatic markers. Scale bars: 15 µm.

To confirm interaction between AT1-AA and dopaminergic neurons and glial cells, we performed fluorescent labeling of AT1-AA previously to culture treatments. We used primary mesencephalic cultures containing both neurons and different types of glial cells, and cultures of different cell lines including rat dopaminergic neurons (N27), human microglia (HMC3) and rat astrocytes (C6). Both in neuron-glia cultures and cultures of different cell lines, we observed fluorescent autoantibodies on neuronal and glial cell membranes, presumably binding AT1 receptors, as observed with double immunofluorescence and confocal microscopy (Fig. 4).

Unfortunately, we do not have a source of ACE2-AA (such as preeclamptic serum for AT1-AA) to get enough quantities of ACE2-AA to be used for similar in vitro or in vivo experiments.

## Discussion

The present results show that levels of serum AT1-AA (AT1 receptor agonists) and ACE2-AA (ACE2 antagonists) are significantly higher in PD patients than in non-PD controls, possibly as a consequence of the dopaminergic degeneration and the corresponding neuroinflammatory changes. Together with age, a well-known risk factor for PD, both AT1-AA and ACE2-AA had a positive association with PD. Both autoantibodies were found in the CSF of four PD patients without additional pathology that had a CSF sample. As only four patients of our cohort had CSF samples, the observations cannot be generalized to the complete PD population. However, the presence of increased levels of autoantibodies in the CSF of each of these patients is consistent with that observed in the PD animal models. In addition, the analysis of CSF from each of these four PD patients suggest that the antibodies can be produced intrathecally at least in some PD patients. However, a higher number of CSF samples is necessary to extent this conclusion to all PD patients. Finally, we confirmed in cultures that the AT1-AA interact with dopaminergic neurons and glial cells and that enhance dopaminergic neuron death via AT1 receptors. This is consistent with previous studies showing the enhancing effect of AT1 receptor activation on neuroinflammation and progression of dopaminergic neuron death^16,33^.

The mechanisms leading to the association of AT1-AA and ACE2-AA with PD remain to be fully clarified. However, in animal models, infusions of IL-6, IL-17 or TNF-α induced an increase in serum levels of AT1-AA, suggesting that proinflammatory cytokines are involved in generation of these autoantibodies, as these cytokines have been reported to increase Tgm2 gene expression (which encodes tissue transglutaminase 2, TG2), and TG2 activity is required for cytokine-induced AT1-AA generation, which is blocked by TG2 inhibitors^35–37^. Among cytokines, TNFSF14 (LIGHT), produced mainly by cells of the immune system, has been found particularly important. First, because LIGHT, acting on inflammatory and structural cells, induces initiation of proinflammatory responses and promotes the production of several inflammatory mediators, such as the above-mentioned TG2-related cytokines^38^. Second, because LIGHT has been shown to induce TG2 production, and the enzyme TG2 produces a posttranslational modification of AT1 receptor protein at the level of its second extracellular loop, which generates a neoantigen that both triggers autoimmune production of AT1-AAs and stabilizes AT1 receptors^23,25,39^. TG2-induced changes interfere AT1 internalization and elimination, leading to increased AT1 expression and AT1 receptor sensitization^24^. Interestingly a recent study observed that TNFSF14 (LIGHT) gene was upregulated in PD patients compared with controls, and it was included in a set differentially expressed genes that may constitute potential gene biomarkers of PD^40^.

**Fig. 5:**
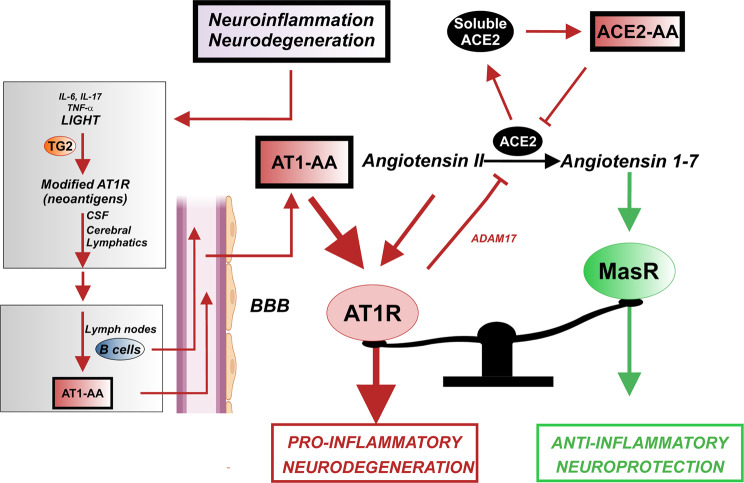
Proposed model for AT1-AA and ACE2-AA generation and effects in PD. Dopaminergic degeneration/neuroinflammation induces an increase in modified AT1 receptors, possibly mediated by inflammation-related cytokines and transglutaminases (TG2), acting as neoantigens that reach the CSF. From the CSF, *via* the lymphatic system, neoantigens can drain into cervical lymph nodes. In the cervical nodes, B cells can respond against neural autoantigens, inducing antigen-specific B cells and circulating autoantibodies. Activated B cells and/or autoantibodies may then migrate into the intrathecal compartment through the BBB, as circulating AT1-AA induce BBB disruption acting on endothelial AT1 receptors. In the brain, AT1-AA enhance AT1 receptor activation and the proinflammatory arm (Ang II/AT1) of the nigral RAS. Furthermore, AT1 activation promote shedding of transmembrane ACE2 into a soluble circulating form by activating proteases such as ADAM17, which decreases local Angiotensin 1–7 and the anti-inflammatory RAS arm (Ang 1–7/MasR) function. The increase in soluble ACE2 levels may induce ACE2-AA, which act as ACE2 antagonists further reducing the activity of transmembrane ACE2 and the anti-inflammatory RAS arm function. Green lines, beneficial effects; red lines, detrimental effects. AT1R: AT1 receptors; AT1-AA: AT1 receptor autoantibodies; ACE2-AA: ACE2 Autoantibodies; BBB: Blood-brain barrier; MasR: Mas receptor. Some elements of this image were modified with permission (license: 5315500675007) from our previous article (DOI: 10.1016/j.jaut.2021.102683) and using BioRender.com (license: SD23YG2KW1).

Mechanisms involved in the increase in serum ACE2-AA are unknown, as upregulation of these autoantibodies has been investigated just in a few studies up to now^41^. It has been suggested that an increase in ACE2-AA may be related to the increase in levels of circulating ACE2^26,30^. This has been related to shedding of ACE2 from the cell surface (i.e. transmembrane ACE2) into a soluble/circulating form. ACE2 shedding has been related to activation of the proteases such ADAM17 (TNF-α converting enzyme) and TMPRSS (transmembrane protease serine 2), which has been intensely studied as mechanisms involved in SARS-CoV-2 infection in COVID-19^42,43^. Activation of the proinflammatory AT1 receptors increases ADAM17 and TMPRSS activity^44,45^. AT1-AA may induce AT1 receptor overactivity, which leads to increased ACE2 shedding and subsequent increase in ACE2-AA (and also increased TNF-α shedding, as ADAM17 is also known as TNF-α converting enzyme) (Fig. 5). Consistent with this, we did not found correlation between serum levels of ACE2-AA and the studied set of interleukins or 27-hydroxycholesterol in the PD or non-PD cohort; however, the results showed a positive and significant relation of ACE2-AA with AT1-AA. A reduction in ACE2 activity at the cell surface (i.e. in transmembrane ACE2) further shifts the balance towards the RAS proinflammatory arm, because ACE2 transforms the proinflammatory Ang II into the anti-inflammatory Ang 1–7. In addition, ACE2-AA may exert a direct antagonistic effect on ACE2. AT1-AA (by agonistic stimulation of AT1 receptors and by subsequent ACE2 shedding) and ACE2-AA antagonistic effects on ACE2 may enhance the RAS proinflammatory responses, neuroinflammation and dopaminergic degeneration (Fig. 5).

We found high levels of AT1-AA and ACE2-AA in the CSF from animal PD models and from 4 PD patients. Although only 4 patients in our cohort had CSF samples, intrathecal synthesis of AT1-AA and ACE2-AA was observed in these patients, suggesting that activated B cells may migrate into the intrathecal compartment followed by clonal expansion leading to intrathecal secretion of these autoantibodies. Interestingly, it is also possible that circulating AT1-AA may contribute to disruption of the BBB in patients, which is consistent with previous studies showing that activation of endothelial AT1 receptors plays a key role in BBB opening^17,18,46^. Hypertension increases BBB permeability, which is blocked by AT1 antagonists and not by other antihypertensive drugs, suggesting that activation of endothelial AT1 receptors is responsible for the increase in BBB permeability and not the hypertension itself^17,18,46^. ACE2-AA can also lead to decrease in ACE2 activity and the subsequent decrease in levels of Ang 1–7 at the BBB, which may also contribute to the increase in BBB permeability^47^. There is growing evidence showing that inflammation-related peripheral processes may initiate or contribute to the progression of neurodegeneration^48,49^, and the above-mentioned observations support that generation of AT1-AA and ACE2-AA induced by dopaminergic degeneration, or by peripheral inflammatory processes, could disrupt BBB facilitating the access of deleterious compounds, the activated B cells, and perhaps autoantibodies to the CNS, contributing to PD progression.

Interestingly, we found significant correlation between serum cytokines and levels of AT1-AA in our cohort of PD patients but not in non-PD controls, suggesting that mechanisms related to PD, particularly PD-related increase in circulating AT1-AA, may contribute to the increase in levels of peripheral inflammatory cytokines by activating AT1 receptors of peripheral cells, which is known to induce production of proinflammatory cytokines in different tissues. This is consistent with the increase in TNF-α levels in cultures treated with AT1-AA in the present experiments. A significant correlation was also observed between serum AT1-AA and 27-hydroxycholesterol (*ρ* = 0.347, *P* < 0.001). Several previous studies have associated serum 27-hydroxycholesterol with cognitive dysfunction and neurodegeneration, particularly in Alzheimer disease, and the involvement of brain RAS in these effects has been suggested^50^.

It is now known that a number of brain diseases are related to pathogenic autoantibodies acting on neuroglial surface proteins, and that CSF from these patients contains autoantigen-specific B cells that may lead to the intrathecal synthesis of these autoantibodies^4,5^. A suggested mechanism of generation and access of AA to brain targets is the following (Fig. 5): cell death (e.g. dopaminergic cell death in PD) and the related neuroinflammatory milieu, may generate soluble neuronal autoantigens (in this case modified AT1 receptors and ACE2) that reach the CSF. From the CSF, neoantigens can drain using the now known cerebral lymphatic system into cervical lymph nodes. In these cervical nodes, B cells can respond against neural autoantigens, inducing antigen-specific B cells, plasma cells and circulating autoantibodies. Activated B cells can then migrate into the intrathecal compartment, followed by clonal expansion, class switching, affinity maturation and differentiation into cells that secrete intrathecal antibodies leading to neuronal damage^4,5^. In fact, a bidirectional movement of B cells between the brain and the cervical lymph nodes across the BBB has been suggested^51^, which may be facilitated in the case of circulating AT1-AA as they can promote BBB disruption (see above). In the case of the present autoantibodies, the results (although from an insufficient number of patients to be generalized to the total population of PD patients) show that autoantibodies can be produced intrathecally in the studied patients. To clarify this point, we investigated whether experimentally induced dopaminergic degeneration can trigger the production of AT1-AA and ACE2-AA. We injected rats with 6-OHDA and looked for the presence of these autoantibodies in serum and/or CSF, and we found a significant increase of circulating and CSF autoantibodies in the 6-OHDA-lesioned rats. In addition, we observed increased levels of LIGHT and transglutaminase activity in the nigral area of 6-OHDA-lesioned rats, which may contribute to transformation of AT1 receptors in neoantigens, as described above. Interestingly, the increase in transglutaminase activity has also been related to the formation of protein aggregates in neurodegenerative diseases, including PD, and neuroinflammation^52^. AT1-AA also induced a marked increase in TNF-α levels in primary cultures, possibly related to the microglial proinflammatory response induced by AT1 activation^34^, which may further enhance AT1-AA production^36^. This indicates that dopaminergic neuron death and the accompanying neuroinflammatory response can induce the generation of autoantibodies that contribute to further progression of dopaminergic degeneration and PD.

In cultures, we confirmed that administration of AT1-AA enhances dopaminergic neuron death, and that this is inhibited by simultaneous treatment with the AT1 antagonist candesartan. The results in cultures confirm that, independent of the possible effects of circulating RAS autoantibodies on BBB permeability, or other possible in vivo mediating factors, AT1-AA can directly exert deleterious effects via AT1 receptor activation in neurons and glial cells. Furthermore, using confocal microscopy and fluorescent labelling of AT1-AA, we showed interactions of autoantibodies with dopaminergic neuron and glial cell surface, presumably binding AT1 receptors.

The deleterious effect of AT1-AA is consistent with several previous studies showing the enhancing effect of AT1 overactivity on dopaminergic degeneration^16,33^, and the neuroprotective effects of drugs such as candesartan and telmisartan in PD animal models and clinical studies^15,16,33^. This is further supported by a recent study showing that the population of dopaminergic neurons most vulnerable to degeneration in PD can be identified by their high expression of the AT1 gen^11^. Interestingly our results in PD patients are consistent with the role of autoimmunity and RAS in PD progression, as we observed a significant increase of these autoantibodies in the PD cohort, and the used logistic model indicated that both immunosuppressive treatments and ACE inhibitors significantly decreased the probability of PD diagnosis, which is consistent with other recent studies also showing a negative association between the use of these drugs and risk of PD diagnosis^53,54^. Our clinical study has some limitations. A first limitation of the study is the relatively low sample size, particularly in the case of CSF samples, which are only exceptionally obtained from PD patients without additional pathologies. The cross-sectional methodology cannot determine causality but association. However, these limitations are compensated by confirmations undertaken in experimental models, which support the clinical observations.

In conclusion, the present results suggest for the first-time autoantibodies against AT1 receptors and ACE2 as a new mechanism of progression of dopaminergic degeneration and neuroinflammation in PD, via activation of the brain RAS proinflammatory axis. These autoantibodies are increased both in serum and CSF. The data suggest that the process of neurodegeneration and neuroinflammation occurring in PD induces the production of AT1-AA and ACE2-AA. Therapeutical strategies blocking the production, or the effects of these autoantibodies (e.g. with ARB drugs) may be useful against the progression of PD.

## Methods

### Clinical studies

A total of 106 adult controls and 117 adult PD patients were included. Controls were recruited from users of a dental clinic at the Primary Health-Care Unit Fontiñas (Santiago de Compostela, Spain). PD patients were recruited from June 2018 to November 2019 at Alvaro Cunqueiro University Hospital Complex (Vigo, Spain). The study was approved by the Galician Drug Research Ethics Committee (CEIm-G), protocol 2017/590, and the research was carried out in accordance with the principles of the Helsinki Declaration. The main demographic and clinical characteristics of PD patients and control subjects at the time point of investigation are reported in Supplementary Table 1. The sample size per group was calculated assuming a medium-size effect (Cohen’s *d* = 0.5)^55^, in Wilcoxon signed-rank test and accepting an alpha risk of 0.05 and a beta risk of 0.1 in a two-sided test. Nonparametric correction based on the lower Pitman Asymptotic Relative Efficiency (~15%) was applied to correct *t* test sample size calculation. It has been anticipated a missing data rate of 5% due to incomplete primary endpoints (AT1-AA). At least 104 subjects per group are needed to detect a difference in means of 0.5 units. This calculation was carried out using the PWR package^56^ in R^57^. Inclusion criteria for the patient group were subjects who had an established diagnosis of PD according to the clinical diagnostic criteria of the United Kingdom Parkinson’s Disease Society Brain Bank^58^ with an age over 40 years, participating voluntarily and who have provided written informed consent. Control group requirements in this group were to not have been diagnosed of PD or other neurodegenerative diseases and to participate voluntarily after signing the informed consent. A total of 121 patients and 114 controls participated in the study, but individuals with comorbidities that are known to alter levels of AT1-AA (liver transplant, APOE4 carrier, multiple sclerosis) or with incomplete information in their medical records were excluded, remaining 117 patients and 106 controls in the final sample.

At the time of recruitment, a blood sample was obtained, and serum was separated by centrifugation at 1500 g for 20 min. Then, aliquots of serum samples were stored at −80 °C until processed for quantification of AT1-AA, ACE2-AA, IL-17, IL-6, TNFSF14 (LIGHT), TNF-α, and 27-OHC levels. PD patients who agreed to participate in the study and that, for other clinical reasons of diagnosis and/or treatment, were subjected to lumbar puncture test, were asked to give a sample of cerebrospinal fluid (CSF) excess to confirm the absence or presence of AT1-AA and ACE2-AA in CSF and study the possibility of intrathecal synthesis of autoantibodies by estimation of corrected antibody index. All patients were followed up using an electronic health information system until July 2021. Clinical data assessing demographics, comorbidities, symptoms, physical and radiological findings, disease stage, treatment and laboratory tests results were collected from electronic medical records.

### Anti-AT1 and anti-ACE2 autoantibody measurements in human serum and CSF

Serum and Cerebrospinal fluid AT1-AA and ACE2-AA levels were measured using two specific solid-phase, sandwich enzyme-linked immunosorbent assays (ELISAs) for the quantitative determination of these autoantibodies (Catalog Number 12000 and 16000, respectively; Cell Trend; Luckenwalde, Germany). Manufacturer’s instructions were followed. Absorbance was measured at 450/620 nm using an Infinite M200 multiwell plate reader (TECAN; 2.0 software) and AT1-AA and ACE2-AA concentrations were quantified using specific standard curves from each one (4PL curve fit). In both cases, samples with values over the standard curve were diluted with assay buffer to get their absorbances within the standard curve.

### Interleukins and 27-hydroxycholesterol quantification in human serum

Levels of IL-17, IL-6; LIGHT; TNF-α and 27-OHC were quantified with human-specific enzyme-linked immunosorbent assay kits according to the manufacturers’ instructions (Cat# BMS2017HS [for IL-17]; Cat# BMS213HS [for IL-6]; Cat# BMS2218 [for LIGHT]; Cat# BMS223HS [for TNF-α] from Invitrogen and LS-F40084 [for 27-OHC] from LSBio).

### Determination of intrathecal synthesis of AT1-AA and ACE2-AA

Quantitative expressions of possible intrathecal synthesis of AT1-AA and ACE2-AA were estimated by calculation of the CSF/serum quotient. Four PD patients were studied for the antibody AT1-AA and AT2-AA in simultaneously collected sera and CSF. The quotients between CSF and serum levels of albumin (Q_ALB_ = Albumin_CSF_[mg/dL]/Albumin_serum_[mg/dL]), total IgG (Q_IgG-Total_ = IgG_CSF_[mg/dL]/IgG_serum_ [mg/dL]), and each specific antibody (Q_AT1-AA_ = IgAT1-AA_CSF_[U/mL]/IgAT1-AA_serum_[U/mL] and Q_ACE2-AA_ = IgACE2-AA_CSF_[U/mL]/IgACE2-AA_serum_[U/mL]) were calculated. Intrathecal production level was evaluated by calculating corrected antibody index (AI) as AI_AT1-AA_ = Q_AT1-AA_/Q_IgG-Total_; AI_ACE2-AA_ = Q_ACE2-AA_/ Q_IgG-Total_ (for Q_IgG-Total_ < Q_LIM_, where Q_LIM_ was estimated as Q_LIM_ = 0.93x(Q_ALB_^2^ + 6 × 10^−6^)^1/2^–1.7 × 10^−3^). Values of AI upper than 1.5 were considered indicative of intrathecal synthesis^31,32,59,60^. Albumin concentration in serum and CSF was estimated using BCG Albumin Assay Kit (Sigma Aldrich, ref. MAK124), following manufacturer’s instructions.

### Effects of brain dopaminergic degeneration on serum and CSF AT1-AA and ACE2-AA levels in rat PD models

All animal experiments were carried out in accordance with the European Communities Council Directive 2010/63/EU 145 and Directive 86/609/EEC and were approved by the corresponding Ethics Committee at the University of Santiago de Compostela (protocol 14715012/2021/012; last revision 16/04/2021). For in vivo experiments, we used young male Sprague–Dawley rats (2–3-month-old) obtained from the Center of Experimental Biomedicine of Galicia (CEBEGA). Animals were housed in conditions of constant room temperature (21–22 °C) and a 12:12 h light-dark cycle and given free access to food and water, and randomly distributed in different experimental groups.

We used a rat 6-OHDA PD model to study if dopaminergic degeneration may contribute to the increase serum and CSF levels of AT1-AA and ACE2-AA. Rats were injected with saline (*n* = 6) or 6-OHDA in the medial forebrain bundle (MFB) and killed 10 days after injection ((*n* = 6; i.e. within the acute period of the lesion) and 4 weeks after injection (*n* = 6; i.e. when the lesion is complete and stabilized). Administration of 6-OHDA was performed in rats deeply anesthetized with a mixture of ketamine (50 mg/Kg) and Medetomidine (0.4 mg/Kg). Thirty minutes prior to 6-OHDA injection, rats were treated with the selective inhibitor for the norepinephrine transporter desipramine (Sigma, 25 mg/kg i.p.) to prevent uptake of 6-OHDA by noradrenergic terminals. Rats were injected in the MFB with 12 μg of 6-OHDA (to provide 8 μg of 6-OHDA free base; Sigma) in 4 μl of sterile saline containing 0.2% ascorbic acid. The stereotaxic coordinates were 3.7 mm posterior to bregma, 1.6 mm lateral to the midline and 8.8 mm ventral to the skull at the midline. The tooth bar was set at −3.3 mm. The solution was injected using a 5-μl Hamilton syringe coupled to a motorized injector (Stoelting), at a rate of 0.5 μl/min; the microsyringe was left in situ 5 min after injection. Control animals were injected with 4 μl of sterile saline alone. Rats were killed by decapitation and blood and CSF from these animals were collected in the moment of sacrifice to look for the presence of autoantibodies. The area of the substantia nigra in the ventral mesencephalon was carefully dissected, and possible mechanisms involved in autoantibody production were studied by enzyme-linked immunosorbent assays (ELISA), analysing levels of TNFSF14 (LIGHT) cytokine and transglutaminase activity in the degenerating substantia nigra tissue.

In addition, stored serum samples (*n* = 10) from a previous study using a different rat PD model and the corresponding controls (*n* = 10) were processed as those from the 6-OHDA lesioned rats (see above). This second PD model was induced by administration of neurospecific adeno-associated viral vectors serotype 9 coding for a mutated form of human alpha-synuclein (SynA53T) and fully characterized and detailed in our previous study^16^.

### TNFSF14/Light and transglutaminase activity in rat ventral midbrain

TNFSF14/Light and transglutaminase activity determination in the substantia nigra of ventral mesencephalon were measured using the specific enzyme-linked immunosorbent assays (ELISA) MBS923672 (MyBioSource, Inc. San Diego, USA) for TNFSF14/Light and the assay Kit ab204700 (Abcam) for transglutaminase activity. In both cases, manufacturer´s instructions were followed.

### Extraction of Blood and cerebrospinal fluid from rats

Blood extraction was performed after the decapitation of the animal in the moment of the sacrifice by decantation. Then, the serum was separated by centrifugation at 2000 g for 10 min. Finally, aliquots of serum samples were stored at −80 °C until processed. Collection of CSF was performed following the method previously described^61^. Briefly, deeply anesthetized rats were positioned in a stereotaxic frame with the head flexed downward at 45° degrees. The superficial and underlying layer of muscles were separated along the midline by blunt dissection and until dura mater exposure. Immediately after the exposure, a 30 G needle attached to 1 ml syringe was inserted at a 30° angle to the dura, from the caudal end of the incision and the aspiration achieved by pulling back the syringe plunger. CSF was immediately stored at −80 °C until processed for quantification of autoantibodies.

### AT1 and ACE2 autoantibody measurements in rat serum and CSF

Serum and CSF AT1-AA and ACE2-AA levels were measured using the same ELISA kits used in humans with a light modification as previously described^62^. The HRP-conjugated secondary antibody used was goat antirat (1:2500, ab97057, Abcam). Immunoreactivity was detected with an Immun-Star HRP Chemiluminescent Kit (170–5044, Bio-Rad; Hercules, CA) and luminescence was measured using an Infinite M200 multiwell plate reader (TECAN).

### Isolation and purification of AT1-AA

AT1-AA were obtained from serum samples of pregnant women who suffered preeclampsia. These women were patients from Obstetric Service of the University Hospital Complex of Santiago de Compostela. All patients were informed of the study’s purpose and protocol and signed an informed consent. Approval for this study was obtained from the Galician Drug Research Ethics Committee (CEIm-G), protocol 2017/618, and the research was carried out in accordance with the principles of the Helsinki Declaration. Isolation of AT1-AA from the serum samples was carried out by affinity chromatography and subsequent titration using a specific enzymatic assay (ELISA)^63,64^. One ml of serum was passed through a specific purification column for anti-human IgG. The AT1-AAs were purified from the human IgG fraction by binding the epitope to the corresponding sequence for the second extracellular loop of the AT1 receptor covalently bound to the gel activated by Sepharose 4B CNBr. Unbound IgG was washed away, and bound IgG was eluted with 200 mM glycine buffer. Eluded IgG were kept in neutralization buffer (3 M TRIS-HCl and 3 M KCl). Final concentration of AT1-AA autoantibody was quantified in a Nanodrop Spectrophotometer 252 (Thermo Fisher Scientific) with nanodrop 2000 v1.4.1 software. Obtained AT1-AA were used for the present studies.

### Effects of AT1-AA on dopaminergic cell death in primary mesencephalic cultures

Rat primary mesencephalic cultures were used to determine the effect of different doses of AT1-AA on the loss of cell viability and levels of the proinflammatory marker TNF-α induced by a low dose of the dopaminergic neurotoxin 6-OHDA, and if AT1-AA effects are blocked by AT1 receptor antagonists such as candesartan, which would confirm that the observed effects are via AT1 receptor activation. Ventral mesencephalic tissue was dissected from rat embryos of 14 days gestation (E14). The tissue was incubated in 0.1% trypsin (Sigma), 0.05% DNase (Sigma), and DMEM (Dulbecco’s Modified Eagle Medium; Gibco, Invitrogen, Paisley, UK) for 20 min at 37 °C and was then washed in DNase and/or DMEM and mechanically dissociated. The resulting cell suspension was centrifuged at 50 g for 5 min, the supernatant removed, and the pellet resuspended in 0.05% DNase/DMEM to the final volume required. The number of viable cells in the suspension was estimated with acridine orange/ethidium bromide, and cells were plated on multiwell plates (12-, 24- or 96-wells; Falcon, Becton Dickinson, Franklin Lakes, NJ, USA) previously coated with poly-L-lysine (100 µg/ml; Sigma) or containing coverslips. The cells were seeded at a density of 1.5 × 10^5^ cells/cm^2^ and maintained in control conditions [DMEM/HAMS F12(1:1) containing 10% fetal bovine serum (FBS; Biochrom KG, Berlin, Germany)]. The primary neuron-glia cultures were maintained in a humidified CO_2_ incubator (5% CO_2_; 37 °C) for 7 days in vitro (DIV); the medium was totally removed on day 2 and replaced with a fresh culture medium.

### Treatment of cultures and Cell Viability

Primary mesencephalic cultures were used to study the effects of AT1-AA on neurodegeneration induced by the dopaminergic 6-OHDA. Cultures were exposed on 6 DIV to 6-OHDA alone (10 µM, based on the results of previous studies;^65^ or AT1-AA (10 and 100 ng/µL) or 6-OHDA plus AT1-AA (10 and 100 ng/µL) or 6-OHDA plus AT1-AA (10 and 100 ng/µL) plus the angiotensin type I receptor blocker candesartan (1 μM; 4791, Tocris) for 24 h. A first series of cultures (*n* = 6 per group) were used to evaluate cell viability using 3-(4,5-dimethylthiazol-2-yl)-2, 5-diphenyltetrazolium bromide (MTT) assay, which is based on the conversion of MTT from yellow to dark blue formazan crystals by mitochondrial dehydrogenases. Briefly, 20 µL MTT (5 mg/ml; Sigma-Aldrich) were added to each well. After incubation at 37 °C for 4 h, the medium and MTT was removed, and the converter dye was solubilized with 50 µL of 0.04 mol/L acidic isopropanol. Finally, the absorbance values were measured with an Infinite M200 multiwell plate reader (TECAN) at 570 nm with a reference wavelength of 690 nm.

### TNF-α levels in primary mesencephalic cultures

In a second series of experiments, cell culture supernatants (n = 6 per group) were collected and centrifuged at 2000 × g for 10 min to eliminate cell debris. Then, the supernatants were used to measure TNF-α levels using the specific Elisa kit KRC3011 from Invitrogen, according to the manufacturer’s instructions.

### TH immunohistochemistry of primary mesencephalic cultures and cell counting

The cultures were fixed with 4% paraformaldehyde in Dulbelcco´s phosphate-buffered saline (DPBS, pH 7.4) for 20 min, and endogenous peroxidase activity was quenched by incubation for 5 min with 3% H_2_O_2_ in DPBS. The cultures were then preincubated with a blocking solution containing 10% normal serum in DPBS with 1% Bovine serum albumin (BSA) and 0.3% Triton X-100 (Sigma) for 1 h. The cultures were then incubated at 4 °C with mouse anti-TH (1:30,000; Sigma, T2928) before being washed and incubated for 1 h with biotinylated horse antimouse (1:500; Cat# BA-2001, Vector, Burlingame, CA). Finally, the cultures were washed again and incubated for 90 min with avidin-biotin-peroxidase complex (ABC, Vector; 1:500). Labeling was revealed with 0.04% H_2_O_2_ and 0.05% 3,3′-diaminobenzidine (Sigma) as a chromogen. Microphotographs were taking in a Nikon FX-III system accommodated to Eclipse TE300 Inverted Microscope (Nikon, Japan).

Effects of different treatments were also quantified by counting TH-immunoreactive neurons as described in our previous studies^33^. Briefly, TH-positive neurons cells were counted (6 wells per treatment in at least 2 independent experiments per treatment) in five randomly chosen longitudinal and transverse microscopic fields along the diameter of the culture dish, away from the curved edge. The operator was blind to the treatment condition. The microscopic field was defined by a 0.20 × 0.20 cm reticule. The average number of TH-positive cells in a control culture dish was 1325 ± 67 (SEM). The results were normalized to the counts of the control group.

### Cultures of N27 dopaminergic neuron, C6 astroglial and HCM3 microglial cell lines

Dopaminergic N27 cell line derived from rat female mesencephalic tissue (SCC048, Millipore, MA, USA) was cultured in RPMI 1640 medium supplemented with 10 % FBS, 2 mM L-glutamine (Sigma), 100 U/ml penicillin, and 100 μg/ml streptomycin.

The C6 astroglial cells (CB_92090409, Sigma) were cultured in Ham’s F12 medium with 10% FBS, 2 mM L-Glutamine (Sigma), 100 U/ml penicillin and 100 μg/ml streptomycin.

The HMC3 microglial cell line, a gift from Dr. Dora Brites (Research Institute for Medicines, University of Lisboa, Portugal), established through SV40-dependent immortalization of a human fetal brain-derived primary microglial culture, was cultured in DMEM medium (Sigma) with 2 mM L-Glutamine (Sigma), 100 U/ml penicillin and 100 μg/ml streptomycin.

All cell line cultures were maintained at 37 °C and 5% CO_2_ in a humidified incubator in a 75 cm^2^ culture flask. Once cells became confluent, they were re-seeded onto 12-well plates (Falcon, Becton Dickinson, Franklin Lakes, NJ, USA), containing glass coverslips, at a density of 0.5 × 10^5^ cells/cm^2^

### Treatment of primary mesencephalic cultures and cell lines with labelled AT1-AA

AT1-AA were labelled with Alexa Fluor® 647 Conjugation kit (ab269823, Abcam) following manufacturer´s instructions. Then, primary mesencephalic cultures and different cell lines were treated with the labelled AT1-AA (100 ng/µL) for 24 h and fixed with 4% paraformaldehyde in Dulbecco’s phosphate-buffered saline (DPBS; pH 7.4) for 20 min. Next, primary cultures and cell lines were processed for fluorescent labelling and incubated overnight at 4 °C with the corresponding primary antibodies diluted in DPBS-1% bovine serum albumin (BSA) with 2% normal donkey serum (Sigma). Astrocytes, microglia, and dopaminergic neurons in primary cultures were identified with the following primary antibodies: rabbit anti glial fibrillary acidic protein (GFAP) as a marker of astrocytes (1:500; Z0334, Agilent Technologies), mouse antirat CD11b (complement receptor-3, clone OX-42; 1:50; MCA275, Bio Rad) as a marker of microglia and mouse anti-tyrosine hydroxylase (TH; 1:10,000; T1299, Sigma) as a marker of dopaminergic neurons. N27, C6 and HMC3 cells were visualized with mouse monoclonal anti-α-tubulin antibody (1:2000; T5168, Sigma). The cultures were rinsed with DPBS and then incubated for 2 h at room temperature with the corresponding secondary antibodies, Alexa Fluor 488-conjugated donkey antimouse IgG (1:200; A21202, Molecular probes) or Alexa Fluor 488-conjugated donkey anti-rabbit IgG (1:200; A21206, Molecular probes). Then, primary cultures and cell lines were incubated for 15 min with the DNA-binding dye Hoechst 33342 (3 × 10^−5^ M in DPBS; Sigma) to visualize cell nuclei and finally coverslipped with Immu-Mount (9990402, Thermo Scientific™ Shandon™). The immunolabeling was visualized by confocal microscopy (TCS SP8; Leica).

### Statistical analysis of the clinical study

Median and interquartile range (IQR) were used as central tendency and dispersion estimators, respectively. Assumptions of normality and homoscedasticity were verified using Anderson Darling test and Fligner-Killeen test, respectively. For quantitative variables, the analysis of two group comparisons were carried on using two tailed t-test when data followed a normal distribution. When normality assumption was rejected, Mann-Whitney U test was used. Welch´s two-samples *t-*test was used for unequal population variances where the assumption of normality is maintained. For categorical variables, the analysis of two group comparisons were carried on using the chi-square test (Pearson’s chi-square test). Spearman’s correlation coefficient was used to study monotonic and linear relationships, respectively. Smaller p-values than 0.05 were considered significant for all the analyses.

A logistic regression was performed to estimate the association between PD and autoantibodies. Confounding variables such as sex, age, immunosuppressive treatment, ACEI, ARAII, smoking habits and heart disease were also included to study the non-casual association. The inclusion of the different predictors was assessed via stepwise model selection using the minimum Akaike information criterion. The estimation of the parameter in the model was adjusted using iterative reweighted least squares. Goodness of fit in logistic regression models was verified using Hosmer-Lemeshow test. Additionally, precision, sensitivity and specificity were used as model performance metrics. All statistical analyses were performed using R^57^.

### Statistical analysis of in vitro and animal experiments

Statistical analyses were performed using SigmaPlot 11.0 (Systat Software, Inc., CA, U.S.A.). Data normality was tested with Kolmogorov–Smirnov test. When the dataset passed the normality test, one-way ANOVA followed by the Student–Newman–Keuls Method for multiple comparisons were used. For nonparametric data, multiple comparisons were carried out by Kruskal–Wallis one-way analysis of variance on ranks test followed by Student–Newman–Keuls. All data were expressed as means ± SEM. Differences were considered statistically significant at P < 0.05. GraphPad Prism 8 software (GraphPad Inc., San Diego, CA, USA) was used to create scatter dot plot graphs.

### Reporting summary

Further information on research design is available in the Nature Research Reporting Summary linked to this article.

## Supplementary information

Supplementary Figures and Tables

## Data Availability

The datasets used and/or analysed during the current study are available from the corresponding author on reasonable request.

## Code availability

Statistical analysis was performed using SigmaPlot 11.0 and R. SigmaPlot and R syntax used for the analysis is available from the corresponding author upon reasonable request.

## References

[1] Tansey, M. G. et al. Inflammation and immune dysfunction in Parkinson disease. *Nat. Rev. Immunol*. 191 (2022). 10.1038/s41577-022-00684-6 PMC8895080 35246670

[2] Sabatino JJ, Probstel AK, Zamvil SS (2019). B cells in autoimmune and neurodegenerative central nervous system diseases. Nat. Rev. Neurosci..

[3] Pruss, H. Autoantibodies in neurological disease. *Nat. Rev. Immunol*. 21, 798–813 (2021). 10.1038/s41577-021-00543-w PMC8111372 33976421

[4] Sun B, Ramberger M, O’Connor KC, Bashford-Rogers RJM, Irani SR (2020). The B cell immunobiology that underlies CNS autoantibody-mediated diseases. Nat. Rev. Neurol..

[5] Negi N, Das BK (2020). Decoding intrathecal immunoglobulins and B cells in the CNS: their synthesis, function, and regulation. Int. Rev. Immunol..

[6] Cabral-Marques O (2018). GPCR-specific autoantibody signatures are associated with physiological and pathological immune homeostasis. Nat. Commun..

[7] Skiba MA, Kruse AC (2021). Autoantibodies as Endogenous Modulators of GPCR Signaling. Trends Pharmacol. Sci..

[8] Riemekasten G, Petersen F, Heidecke H (2020). What Makes Antibodies Against G Protein-Coupled Receptors so Special? A Novel Concept to Understand Chronic Diseases. Front. Immunol..

[9] Labandeira-Garcia JL (2013). Dopamine-angiotensin interactions in the basal ganglia and their relevance for Parkinson’s disease. Mov. Disord..

[10] Wright JW, Harding JW (2019). Contributions by the Brain Renin-Angiotensin System to Memory, Cognition, and Alzheimer’s Disease. J. Alzheimers Dis..

[11] Kamath T (2022). Single-cell genomic profiling of human dopamine neurons identifies a population that selectively degenerates in Parkinson’s disease. Nat. Neurosci..

[12] Jackson, L., Eldahshan, W., Fagan, S. C. & Ergul, A. Within the Brain: The Renin Angiotensin System. *Int. J. Mol. Sci*. **19**, 876 (2018). 10.3390/ijms19030876 PMC5877737 29543776

[13] Labandeira-Garcia JL, Valenzuela R, Costa-Besada MA, Villar-Cheda B, Rodriguez-Perez AI (2021). The intracellular renin-angiotensin system: Friend or foe. Some light from the dopaminergic neurons. Prog. Neurobiol..

[14] Yan R (2020). Structural basis for the recognition of SARS-CoV-2 by full-length human ACE2. Science.

[15] Jo Y, Kim S, Ye BS, Lee E, Yu YM (2022). Protective Effect of Renin-Angiotensin System Inhibitors on Parkinson’s Disease: A Nationwide Cohort Study. Front. Pharmacol..

[16] Rodriguez-Perez AI (2018). Angiotensin Type 1 Receptor Antagonists Protect Against Alpha-Synuclein-Induced Neuroinflammation and Dopaminergic Neuron Death. Neurotherapeutics.

[17] Fleegal-DeMotta MA, Doghu S, Banks WA (2009). Angiotensin II modulates BBB permeability via activation of the AT(1) receptor in brain endothelial cells. J. Cereb. Blood Flow. Metab..

[18] Santisteban MM (2020). Endothelium-Macrophage Crosstalk Mediates Blood-Brain Barrier Dysfunction in Hypertension. Hypertension.

[19] Campbell N, LaMarca B, Cunningham MW (2018). The Role of Agonistic Autoantibodies to the Angiotensin II Type 1 Receptor (AT1-AA) in Pathophysiology of Preeclampsia. Curr. Pharm. Biotechnol..

[20] Fu ML (2000). Autoantibodies against the angiotensin receptor (AT1) in patients with hypertension. J. Hypertens..

[21] Dragun D (2005). Angiotensin II type 1-receptor activating antibodies in renal-allograft rejection. N. Engl. J. Med..

[22] Meyer LS, Gong S, Reincke M, Williams TA (2020). Angiotensin II Type 1 Receptor Autoantibodies in Primary Aldosteronism. Horm. Metab. Res..

[23] Liu, C. et al. Elevated Transglutaminase Activity Triggers Angiotensin Receptor Activating Autoantibody Production and Pathophysiology of Preeclampsia. *J. Am. Heart Assoc*. **4**, e002323 (2015). 10.1161/JAHA.115.002323 PMC4845265 26675250

[24] Liu C (2019). Tissue Transglutaminase-Mediated AT1 Receptor Sensitization Underlies Pro-inflammatory Cytokine LIGHT-Induced Hypertension. Am. J. Hypertens..

[25] Liu C (2014). Tissue transglutaminase contributes to the pathogenesis of preeclampsia and stabilizes placental angiotensin receptor type 1 by ubiquitination-preventing isopeptide modification. Hypertension.

[26] McMillan P, Dexhiemer T, Neubig RR, Uhal BD (2021). COVID-19-A Theory of Autoimmunity Against ACE-2 Explained. Front. Immunol..

[27] Miziolek B (2021). The prevalence and role of functional autoantibodies to angiotensin-converting-enzyme-2 in patients with systemic sclerosis. Autoimmunity.

[28] Arthur JM (2021). Development of ACE2 autoantibodies after SARS-CoV-2 infection. PLoS One.

[29] Miedema J (2021). Antibodies Against Angiotensin II Receptor Type 1 and Endothelin A Receptor Are Associated With an Unfavorable COVID19 Disease Course. Front. Immunol..

[30] Rodriguez-Perez AI (2021). Autoantibodies against ACE2 and angiotensin type-1 receptors increase severity of COVID-19. J. Autoimmun..

[31] Reiber H (1998). Cerebrospinal fluid-physiology, analysis and interpretation of protein patterns for diagnosis of neurological diseases. Mult. Scler..

[32] Reiber H, Lange P (1991). Quantification of virus-specific antibodies in cerebrospinal fluid and serum: sensitive and specific detection of antibody synthesis in brain. Clin. Chem..

[33] Rodriguez-Pallares J (2008). Brain angiotensin enhances dopaminergic cell death via microglial activation and NADPH-derived ROS. Neurobiol. Dis..

[34] Borrajo A, Rodriguez-Perez AI, Diaz-Ruiz C, Guerra MJ, Labandeira-Garcia JL (2014). Microglial TNF-alpha mediates enhancement of dopaminergic degeneration by brain angiotensin. Glia.

[35] Dhillion P (2012). IL-17-mediated oxidative stress is an important stimulator of AT1-AA and hypertension during pregnancy. *Am*. J. Physiol. Regul. Integr. Comp. Physiol..

[36] Irani RA (2010). Autoantibody-mediated angiotensin receptor activation contributes to preeclampsia through tumor necrosis factor-alpha signaling. Hypertension.

[37] Lamarca B (2011). Hypertension in response to IL-6 during pregnancy: role of AT1-receptor activation. Int. J. Interferon Cytokine Mediat. Res..

[38] Herro R, Croft M (2016). The control of tissue fibrosis by the inflammatory molecule LIGHT (TNF Superfamily member 14). Pharmacol. Res..

[39] Liu C, Kellems RE, Xia Y (2017). Inflammation, Autoimmunity, and Hypertension: The Essential Role of Tissue Transglutaminase. Am. J. Hypertens..

[40] Jiang F, Wu Q, Sun S, Bi G, Guo L (2019). Identification of potential diagnostic biomarkers for Parkinson’s disease. FEBS Open Bio..

[41] Takahashi Y, Haga S, Ishizaka Y, Mimori A (2010). Autoantibodies to angiotensin-converting enzyme 2 in patients with connective tissue diseases. Arthritis Res. Ther..

[42] Hoffmann M (2020). SARS-CoV-2 Cell Entry Depends on ACE2 and TMPRSS2 and Is Blocked by a Clinically Proven Protease Inhibitor. Cell.

[43] Lambert DW (2005). Tumor necrosis factor-alpha convertase (ADAM17) mediates regulated ectodomain shedding of the severe-acute respiratory syndrome-coronavirus (SARS-CoV) receptor, angiotensin-converting enzyme-2 (ACE2). J. Biol. Chem..

[44] Deshotels MR, Xia H, Sriramula S, Lazartigues E, Filipeanu CM (2014). Angiotensin II mediates angiotensin converting enzyme type 2 internalization and degradation through an angiotensin II type I receptor-dependent mechanism. Hypertension.

[45] Pedrosa MA (2021). Experimental data using candesartan and captopril indicate no double-edged sword effect in COVID-19. Clin. Sci. (Lond.)..

[46] Bloch S, Obari D, Girouard H (2015). Angiotensin and neurovascular coupling: beyond hypertension. Microcirculation.

[47] Wu J, Zhao D, Wu S, Wang D (2015). Ang-(1-7) exerts protective role in blood-brain barrier damage by the balance of TIMP-1/MMP-9. Eur. J. Pharmacol..

[48] Cunningham C (2013). Microglia and neurodegeneration: the role of systemic inflammation. Glia.

[49] Varatharaj A, Galea I (2017). The blood-brain barrier in systemic inflammation. Brain Behav. Immun..

[50] Mateos L (2011). Upregulation of brain renin angiotensin system by 27-hydroxycholesterol in Alzheimer’s disease. J. Alzheimers Dis..

[51] Palanichamy A (2014). Immunoglobulin class-switched B cells form an active immune axis between CNS and periphery in multiple sclerosis. Sci. Transl. Med..

[52] Gatta NG, Cammarota G, Gentile V (2016). Possible roles of transglutaminases in molecular mechanisms responsible for human neurodegenerative diseases. AIMS Biophysics.

[53] Racette BA (2018). Immunosuppressants and risk of Parkinson disease. Ann. Clin. Transl. Neurol..

[54] Visanji NP (2021). Using artificial intelligence to identify anti-hypertensives as possible disease modifying agents in Parkinson’s disease. Pharmacoepidemiol. Drug Saf..

[55] Cohen, J. *Statistical power analysis for the behavioral sciences*. (Lawrence Erlbaum Associates, 1988).

[56] Champely, S. pwr, Basic Functions for Power Analysis. *R Package Version* 1.3-0 https://CRAN.R-project.org/package=pwr (2020).

[57] R Core Team. R: A language and environment for statistical computing. *R Foundation for Statistical Computing* https://www.R-project.org/ (2020).

[58] Hughes AJ, Daniel SE, Kilford L, Lees AJ (1992). Accuracy of clinical diagnosis of idiopathic Parkinson’s disease: a clinico-pathological study of 100 cases. J. Neurol. Neurosurg. Psychiatry.

[59] Akaishi T (2021). Difference in the Source of Anti-AQP4-IgG and Anti-MOG-IgG Antibodies in CSF in Patients With Neuromyelitis Optica Spectrum Disorder. Neurology.

[60] Jarius S (2020). Cerebrospinal fluid findings in patients with myelin oligodendrocyte glycoprotein (MOG) antibodies. Part 2: Results from 108 lumbar punctures in 80 pediatric patients. J. Neuroinflammation.

[61] Pegg CC, He C, Stroink AR, Kattner KA, Wang CX (2010). Technique for collection of cerebrospinal fluid from the cisterna magna in rat. J. Neurosci. Methods.

[62] Zhang SL (2010). Endothelial dysfunction induced by antibodies against angiotensin AT1 receptor in immunized rats. Acta Pharmacol. Sin..

[63] LaMarca B (2011). Hypertension in response to placental ischemia during pregnancy: role of B lymphocytes. Hypertension.

[64] Wang HP (2014). Exposure to AT1 receptor autoantibodies during pregnancy increases susceptibility of the maternal heart to postpartum ischemia-reperfusion injury in rats. Int. J. Mol. Sci..

[65] Rodriguez-Pallares J (2007). Mechanism of 6-hydroxydopamine neurotoxicity: the role of NADPH oxidase and microglial activation in 6-hydroxydopamine-induced degeneration of dopaminergic neurons. J. Neurochem..

