# Supplementary material for: Angiotensin type-1 receptor and ACE2 autoantibodies in Parkinson´s disease
Source: NPJ Parkinsons Dis. 2022 Jun 14;8:76. doi: 10.1038/s41531-022-00340-9 (PMC9198025; doi:10.1038/s41531-022-00340-9)
Supplement: Supplementary file 1 — Supplementary Figures and Tables [file 41531_2022_340_MOESM1_ESM.pdf]

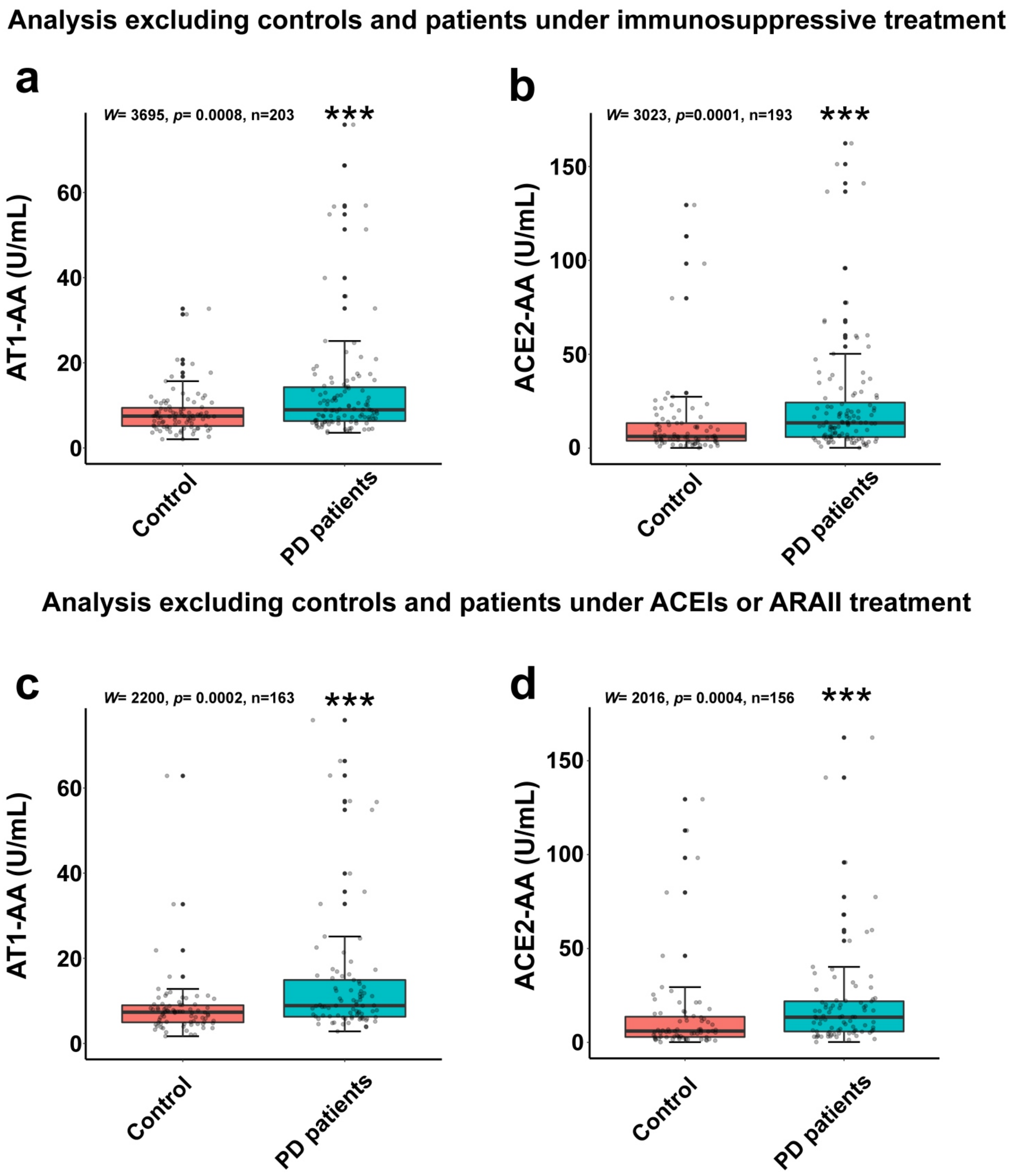

Supplementary Figure 1. Autoantibody serum levels in controls and PD patients excluding those under immunosuppressive treatment (a, b) or ACEIs/ARAII (c, d). Parkinson´s disease (PD) patients had significantly higher serum levels of AT1-AA (**a**, **c**) and ACE2-AA (**b**, **d**). Wilcoxon tests. Data distribution is shown using a box plot with boxes representing the IQR and the median (black line) and whiskers representing ±1.5 IQR. IQR: Interquartile range; AT1-AA: Autoantibodies for AT1 receptors; ACE2-AA: ACE2 Autoantibodies.

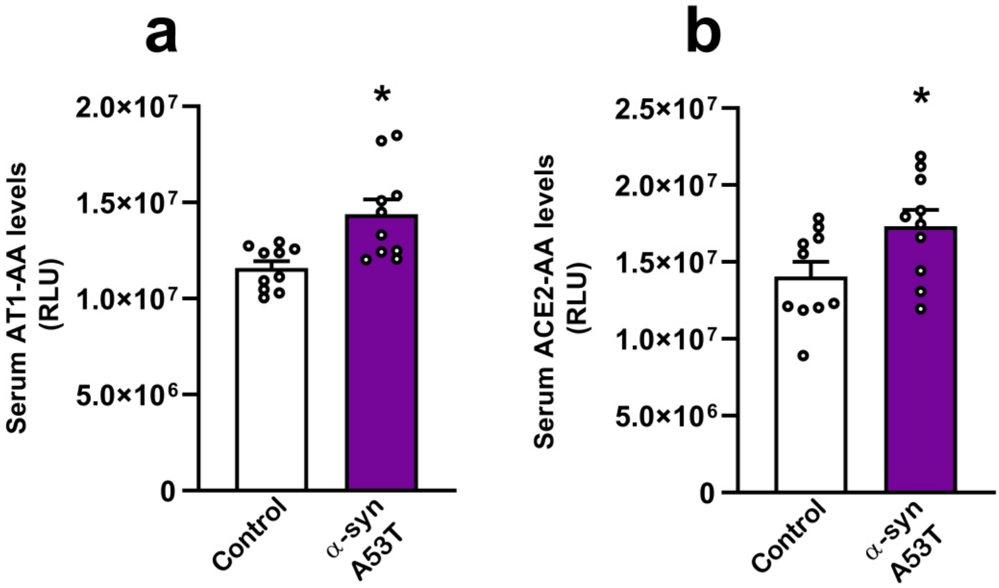

Supplementary Figure 2. Increase in serum AT1-AA and ACE2-AA levels induced by dopaminergic degeneration in rats. Serum levels of AT1-AA (**a**) and ACE2-AA (**b**) 4 weeks after injection of neurospecific adeno-associated viral vectors coding for a mutated form of human alpha-synuclein (α-syn A53T). A significant increase in AT1-AA and ACE2-AA serum levels is observed in rats with dopaminergic lesions. Data are given as means ± SEM. *P<0 .05 compared to the control group. Student–t tests. AT1-AA: Autoantibodies for AT1 receptors; ACE2-AA: ACE2 Autoantibodies.

Supplementary Table 1. Main clinical features of the study subjects.

	Control (n=106)	Patients (n=117)	Total (n=213)	p-value
AT1-AA (U/mL)				
Mean (SD)	8.922 (7.294)	13.505 (13.639)	11.326 (11.297)	p=0.002 (1)
Median	7.556	8.923	8.034	
IQR	4.590	7.969	5.806	
Q1, Q3	5.109, 9.699	6.271, 14.240	5.951, 11.757	
ACE2-AA (U/mL)				
Mean (SD)	13.157 (21.300)	26.872 (50.527)	20.690 (40.583)	p< 0.001 (2)
Median	6.305	13.519	9.985	
IQR	10.084	20.602	15.304	
Q1, Q3	3.265, 13.349	5.974, 26.576	4.800, 20.104	
AGE (years)				
Mean (SD)	64.901 (9.249)	69.430 (10.095)	67.288 (9.944)	p< 0.001 (3)
Median	64.692	70.056	67.311	
IQR	10.561	14.039	12.919	
Q1, Q3	59.286, 69.847	63.078, 77.117	61.074, 73.992	
GENDER				
Man	51 (48.1%)	57 (48.7%)	108 (48.4%)	p=0.928 (4)
Woman	55 (51.9%)	60 (51.3%)	115 (51.6%)	
HYPERTENSION				
No	70 (66.7%)	64 (54.7%)	134 (60.4%)	p=0.069 (4)
Yes	35 (33.3%)	53 (45.3%)	88 (39.6%)	
HEART DISEASE				
No	84 (81.6%)	93 (80.2%)	177 (80.8%)	p=0.796 (4)
Yes	19 (18.4%)	23 (19.8%)	42 (19.2%)	
DIABETES				
No	79 (75.2%)	94 (80.3%)	173 (77.9%)	p=0.360 (4)
Yes	26 (24.8%)	23 (19.7%)	49 (22.1%)	
OBESITY				
No	42 (40.0%)	51 (43.6%)	93 (41.9%)	p=0.588 (4)
Yes	63 (60.0%)	66 (56.4%)	129 (58.1%)	
27-Hydroxycholesterol (27-OHC; ng/mL)				
Mean (SD)	436.658 (208.822)	498.724 (312.173)	469.684 (270.009)	p=0.323 (1)
Median	397.320	413.299	407.360	
IQR	259.707	304.755	277.595	
Q1, Q3	274.495, 534.202	305.888, 610.642	282.580, 560.175	
IL-6 (pg/mL)				
Mean (SD)	1.029 (1.120)	1.224 (1.473)	1.133 (1.320)	p=0.262 (1)
Median	0.670	0.825	0.760	
IQR	0.890	1.138	1.005	
Q1, Q3	0.343, 1.232	0.382, 1.520	0.352, 1.357	
IL-17 (pg/mL)				
Mean (SD)	11.016 (17.354)	11.849 (17.880)	11.461 (17.601)	p=0.429 (1)

Median	3.970	5.155	4.530	
IQR	12.560	15.538	13.810	
Q1, Q3	0.230, 12.790	0.230, 15.768	0.230, 14.040	
TNF-α (pg/mL)				
Mean (SD)	0.637 (0.777)	0.787 (1.367)	0.717 (1.130)	p=0.880 (1)
Median	0.310	0.310	0.310	
IQR	0.450	0.317	0.382	
Q1, Q3	0.310, 0.760	0.310, 0.627	0.310, 0.692	
LIGHT (pg/mL)				
Mean (SD)	1199.294 (1262.424)	1075.585 (1217.903)	1133.467 (1237.610)	p=0.391 (1)
Median	710.760	541.960	629.575	
IQR	2040.345	1709.242	1874.798	
Q1, Q3	134.395, 2174.740	68.683, 1777.925	100.665, 1975.463	
WEIGHT (Kg)				
Mean (SD)	78.027 (14.432)	74.785 (13.965)	76.318 (14.248)	p=0.092 (5)
Median	79.500	73.200	74.750	
IQR	22.250	19.000	18.625	
Q1, Q3	67.000, 89.250	65.000, 84.000	66.375, 85.000	

ACE2-AA: ACE2 autoantibodies; ACEi: Angiotensin converting enzyme inhibitors; AT1-AA: Autoantibodies for AT1 receptors; IQR: Interquartile range; SD: Standard deviation. (1) Wilcoxon rank sum test with continuity correction, Anderson Darling, (AD) test= 0; (2) Wilcoxon rank sum exact test, AD test= 0; (3) Two Sample t-test, AD test= 0.216, Fligner-Killeen (FK) test= 0.444; (4) Pearson's Chi-squared test; (5) Two Sample t-test, AD test= 0.843, FK test= 0.149

Supplementary Table 2. Main clinical features of the study subjects. Baseline characteristics of the PD patients.

	TOTAL PD	GENDER	
		**Males** n=57 (48.7%)	**Females** n=60 (51.3%)
**Age** (years)			
Mean (SD)	69.430 (10.095)	67.60 (11.281)	71.17 (8.559)
Median	70.06	68.62	71.04
IQR	14.33	13.93	11.99
Q1, Q3	63.06, 77.39	61.00, 74.93	66.02, 78.01
**Duration of disease** (years)			
Mean (SD)	7.050 (5.181)	6.312 (3.833)	7.751 (6.149)
Median	6	5.714	7
IQR	6.649	4.938	8.102
Q1, Q3	9.649, 3.00	3.327, 8.265	2.680, 10.782
**Hoehn-Yahr scale**			
Mean (SD)	2.308 (0.715)	2.202 (0.660)	2.408 (0.756)
Median	2	2	2
**UPDRS3 ON**			
Mean (SD)	27.24 (12.74)	27.510 (13.5648)	26.980 (11.993)
Median	26	27	26
IQR	18.75	19.50	18
Q1, Q3	17.75, 36.50	17.50, 37.00	18.50, 36.50
**UPDRS3 OFF**			
Mean (SD)	39.662 (16.133)	39.088 (15.272)	40.235 (17.162)
Median	37	37	40.50
IQR	21.25	16.80	26.75
Q1, Q3	28.25, 49.75	29.75, 46.50	26.00, 52.75
**Cognitive impairment** (%):			
No	68 (58.1%)	34 (59.6%)	34 (56.7%)
Mild	32 (27.4%)	13 (22.8%)	19 (31.7%)
Yes	17 (14.5%)	10 (17.5%)	7 (11.7%)
**MMSE score**			
Mean (SD)	25.37 (4.989)	25.75 (4.814)	25.02 (5.165)
Median	27	27	27
IQR	3	2.75	4.5
Q1, Q3	25, 28	25.25, 28.00	23.50, 28.00
**MoCA score**			
Mean (SD)	21.93 (5.495)	22.05 (5.539)	21.80 (5.499)
Median	23	23	23
IQR	7	7	7
Q1, Q3	19.00, 26.00	19.00, 26.00	19.00, 26.00
**MoCA scalar score*** (0-18)			
Mean (SD)	9.96 (3.018)	9.87 (2.913)	10.06 (3.147)
Median	10	10	10
IQR	4	3	4
Q1, Q3	8.00, 12.00	8.00, 11.00	8.00, 12.00

**Number of antiparkinsonian medications**			
Mean (SD)	2.59 (1.339)	2.66 (1.325)	2.52 (1.359)
Median	2	3	2
IQR	1.75	2	1
Q1, Q3	2, 3.75	2, 4	2, 3
**Levodopa equivalent daily dose**			
(mg)	706 (415.720)	744.132(453.010)	669.391 (377.182)
Mean (SD)	710	756	650
Median	608	595	647.50
IQR	387, 995	400.00, 995.00	343.75, 991.25
Q1, Q3			
**Use of dopamine agonists** (%)	59 (50.9%)	28 (50%)	31 (51.7%)
**Motor fluctuations** (%)	72 (62.1%)	36 (63.2%)	36 (61.0%)
**NMSS score**			
Mean (SD)	69.391 (41.89)	65.070 (38.875)	73.638 (44.599)
Median	62	59	67
IQR	59	38	69.75
Q1, Q3	37.00, 96.00	38.50, 76.50	36.50, 106.25

Baseline characteristics of the PD patients. No significant differences were detected between males and females. UPDRS: Unified Parkinson Disease Rating Scale; MMSE: Mini-Mental State Examination; MoCA: Montreal Cognitive Assessment; NMSS: Non-Motor Symptoms Scale. *adjusted by age and level of education for Spanish population (10-18: no cognitive impairment; 7-9: mild cognitive impairment; <7: cognitive impairment) (Ojeda et al, 2016).

Ojeda, N., Del Pino, R., Ibarretxe-Bilbao, N., Schretlen, D. J., & Pena, J. (2016). Test de evaluacion cognitiva de Montreal: normalizacion y estandarizacion de la prueba en poblacion española [Montreal Cognitive Assessment Test: normalization and standardization for Spanish population]. Revista de neurologia, 63(11), 488–496
